# Chronic Epstein-Barr viral load carriage after pediatric organ transplantation

**DOI:** 10.3389/fped.2024.1335496

**Published:** 2024-01-31

**Authors:** Masaki Yamada, Sharon F. Chen, Michael Green

**Affiliations:** ^1^ National Center for Child Health and Development (NCCHD), Tokyo, Japan; ^2^ Department of Pediatrics, Stanford University, Palo Alto, CA, United States; ^3^ Departments of Pediatrics and Surgery, University of Pittsburgh, Pittsburgh, PA, United States

**Keywords:** pediatrics, solid organ transplantation, Epstein-Barr virus, PTLD, chronic high load carrier

## Abstract

Epstein-Barr virus (EBV) infection and EBV-associated post-transplant lymphoproliferative disorder (EBV/PTLD) is one of the most devastating complications occurring in pediatric solid organ transplant (SOT) recipients. Observations of SOT recipients undergoing serial EBV monitoring to inform reduction of immune suppression to prevent EBV-/PTLD has identified patients who maintain chronic high EBV load (CHL) in their blood. The CHL carrier state has been seen more commonly in pediatric compared to adult transplant recipients. Some but not all CHL may progress to EBV/PTLD. However, little is known regarding the biology of this CHL carrier state and the optimal clinical approach to CHL has not been established. This review summarizes the current knowledge and evidence of chronic high EBV load and introduces commonly adopted approaches from experts in this field.

## Introduction

Epstein-Barr virus (EBV) is a ubiquitous gamma-herpesvirus that establishes life-long latent infection following acute infection in humans. After the immune response controls primary EBV infection, EBV lytic infection occasionally occurs in pharyngeal epithelial cells but progression to lytic infection is uncommon in peripheral EBV-infected B-lymphocytes. Instead, EBV-infected B cells generally maintain latent infection mediated by limited EBV gene expression patterns. However, these latently infected B cells can escape from immune control and proliferate aggressively due to the presence of viral transcripts that stimulate the cell cycle and prevent apoptosis. In immunocompetent individuals, proliferation of EBV-infected B-cells is usually controlled by their T-cell immune response preventing progression to clinical disease. In this context, EBV establishes an equilibrium of persistent infection controlled by the host's immune system although the balance can be transiently disrupted by the presence of intercurrent illnesses or infections. During these periods of disrupted equilibrium, intermittent DNAemia, as defined by nucleic acid amplification testing (NAAT), may occur until control is re-established by the host T-cell response. Clinical disease does not typically develop and the EBV load may not be high enough to measure (1). In the immunosuppressed solid organ transplant (SOT) recipient, the establishment of this equilibrium may be impaired, and disruptions may be persistent, which sometimes can lead to detrimental outcomes such as EBV-associated post-transplant lymphoproliferative disorder (PTLD), although all PTLDs are associated with EBV.

Measurement of EBV loads in the peripheral blood was introduced into clinical care of SOT recipients in the 1990s. Longitudinal observations in these patients have shown that a high EBV load in the peripheral blood is sensitive but not specific for the presence of EBV disease including PTLD (EBV/PTLD) (2). While a high level of EBV DNAemia is typically present in SOT recipients with EBV/PTLD, not all patients with high EBV loads develop disease. The meaning of a given EBV load must be interpreted based on local experience with individual assays given variations in NAAT performance between centers as well as differences attributable to which blood compartment is being measured (e.g., whole blood, plasma) (2). Despite these limitations, the use of sequential surveillance measurement of EBV loads in pediatric SOT recipients has been associated with declining rates of EBV/PTLD, and EBV monitoring is now recommended for SOT recipients at risk for EBV/PTLD (3). An unexpected outcome of surveillance monitoring was recognition of a frequently observed pattern of persistently high EBV loads in otherwise asymptomatic children, raising questions as to the meaning and management of this EBV chronic high load (CHL) carrier state in this population.

## Case

**Figure 1:**
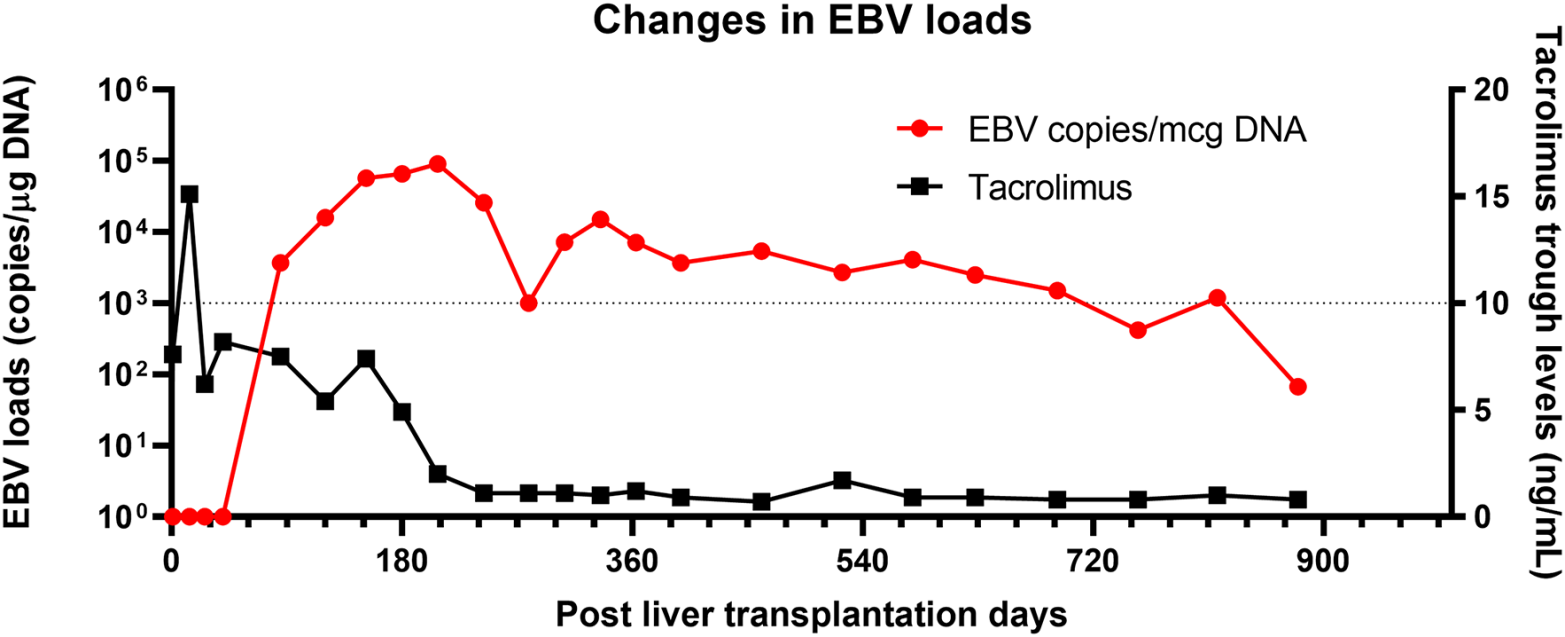
Changes in EBV load in a case with chronic high EBV load carrier. A red line represents EBV load (Left Y axis), and a black line represents tacrolimus trough levels. Tacrolimus was the only immunosuppression therapy the case received during the long-term follow-up. Despite minimal immunosuppression therapy, high EBV load persisted for more than three years.

A 5-month-old girl with a history of biliary atresia and a failed Kasai procedure underwent living-donor liver transplantation (LT). Her cytomegalovirus (CMV) and EBV serostatus were both mismatched (D + R−). She received routine methylprednisolone induction followed by rapid taper and maintenance tacrolimus and prednisolone per clinical protocol. Her postoperative course was complicated with moderate to severe acute cellular rejection on postoperative day (POD) 15 that was successfully treated with additional high-dose methylprednisolone therapy. Subsequently, her post-transplant course was uneventful. She was maintained on oral tacrolimus with a trough goal of 5–10 ng/ml, and prednisone was tapered off 3 months after LT. Of note, routine EBV and CMV load surveillance confirmed onset of her primary EBV infection on POD 85. The EBV load rapidly increased and persisted at a “high” level (1,000 copies/*μ*g DNA for this assay) despite reducing immunosuppression, including minimizing her tacrolimus dose. She maintained high EBV loads for the next six months despite lower target tacrolimus through levels of 3–5 ng/ml. No concerns for rejection were noted. At this point, she met the criteria for a CHL carrier. She remained asymptomatic with good growth and development. Her CHL carrier state persisted for the next 2 years before beginning to spontaneously resolve (Figure 1).

The highlights of this case include (1) the presence of multiple risk factors for the post-transplant CHL carrier state, such as young age at LT and EBV serology mismatch; (2) the child remained asymptomatic throughout the entire CHL carrier state time period; (3) the CHL persisted for more than 2 years despite a timely reduction of immunosuppression and then gradually resolved; (4) and finally, there were no consequences of the CHL carrier state in this patient.

## Definition of CHL

The definition of CHL varies amongst studies. The original definition of CHL was proposed in a study of pediatric heart transplant recipients undergoing EBV load surveillance with whole blood or peripheral blood lymphocyte assays at a single center (4). The definition was met when asymptomatic patients had levels above the threshold where EBV/PTLD historically occurred on their center's assay on at least 50% of samples over a minimum of six months, whether after onset of asymptomatic infection or complete resolution of symptomatic EBV disease, including PTLD (4). Additional studies were also done on liver, intestine, and kidney recipients from the same center using the same definition and assays, allowing comparisons between different organ recipients (5–7). In an effort to support conducting multicenter studies, the North American Pediatric Renal Transplant Consortium subsequently, proposed defining “high-level carriers” as patients with “more than 100 times the detection limit” for EBV loads in blood, regardless of assay, that persisted for more than 6 months (8). There are other studies using different definitions of CHL. However, these did not always assess for a correlation between the CHL carrier state and frequency of progression to EBV/PTLD (Table 1) (8). Other investigators published on chronic EBV load carriers, with some focusing on patients with “high loads” and others on any viral loads with varying definitions. In order to standardize the intensities of EBV loads and achieve risk stratification, an EBV WHO standard was introduced in 2016. However, a threshold for “high” based on the EBV WHO international standard has not been established. Therefore, investigating the epidemiology of CHL remains a challenge across different centers (20).

**Table 1:** Definitions of chronic (persistent) high load carrier state in the literature.

Published year	Report	Cohort	Definition of CHL by viral load and duration	Incidence of PTLD among CHL
2000	Qu et al. (9)	H + K + Li + Lu + SI, Peds	>200 copies/10^5^ lymphocytes >2 months	Not assessed
2003	Campe et al. (10)	K, Peds	>600 copies/10^5^ lymphocytes, DND	Not assessed
2008	Bingler et al. (4)	H + Lu, Peds	>16,000 copies/ml Or >200 copies/10^5^ PBMC ≥50% of samples over at least 6 months	9/20 (45%)
2009	Green et al. (5)	Li, Peds		1/35 (3%) developed PTLD
2010	Lau et al. (6)	SI, Peds		4/35 (11%) developed PTLD
2010	Gotoh et al. (11)	Li, Peds	>5,000 copies/ml	Not assessed
2011	Ishihara et al. (12)	K, Peds	>1,000 copies/µgDNA for over 6 months	Not assessed
2011	Tanaka et al. (13)	K, Peds	>1,000 copies/µgDNA for over 6 months	Not assessed
2013	Höcker et al. (14)	K, Peds	≥10,000 copies/ml, persistent (>50% of samples) EBV load.	1/18 (6%)
2014	Moudgil et al. (8)	K, Peds	>100 times detection limit for each assay ≥2 positive VL or ≥2/3 of measured VL positive over 6 months	7/85 (8%) developed PTLD
2014	Shakibazad et al. (14)	Li, Peds	1,000 copies/ml, DND	Not assessed
2017	Kullberg-Lindh et al. (16)	Li, Peds	≥4.2 log10 Geq/ml >50% of samples for ≥6 months.	0/10 (0%)
2017	Kamei et al. (17)	Li, Peds	>10 000 IU/ml, for ≥6 months	0/31 (0%)
2018	Yamada et al. (7)	K, Peds	Same as reports 4–6.	0/16 (0%)
2020	Ladfors et al. (18)	K, Peds	≥4.2 log10 Geq/ml in >50% of the samples during ≥6 month	0/14 (0%)
2023	Yamada et al. (19)	Li, Peds	1,000 copies/μg DNA, for ≥6 months	0/61 (0%)

H, heart transplant recipients; K, kidney transplant recipients; Li, liver transplant recipients; Lu, lung transplant recipients; SI, small intestinal transplant recipients; CHL, chronic high load; PBMC, peripheral blood nuclear cells; Peds, pediatric patients, DND, duration not defined.

## Incidence and outcome of CHL carrier state in different organ recipients

Both the incidence of the CHL carrier state and progression to PTLD amongst CHL carriers varies by transplanted organ. Results from a single pediatric transplant center using the same definition and EBV PCR assays revealed a CHL carrier incidence of 8% in kidney, 18% in liver, 21% in intestine, and 28% in heart recipients over the first several years post-transplantation. The incidence of PTLD in CHL carriers by organ type in these initial series was 0/16 (0%) in kidney, 1/36 (3%) in liver, 4/35 (11%) in intestine, and 9/20 (45%) in heart transplant recipients (4–7). The markedly higher rate of progression to EBV/PTLD amongst the heart transplant recipients suggests the presence of a different immunopathology associated with the CHL carrier state in heart recipients, which has been investigated in one study (21).

## Biology of CHL carrier state

### What does the presence of a CHL carrier state infer?

Despite more than 20 years of wide usage of EBV load monitoring, uncertainty remains as to the biological meaning of the CHL carrier state. Whether the presence of a persistent high EBV reflects proliferation of EBV infected B cells in tissue, the peripheral blood or both remains uncertain. However, recent work using single-cell-based analysis has revealed that EBV load in whole blood strongly correlates with the frequency of EBV-infected cells in the peripheral blood. Thus, an EBV load likely reflects the number of EBV infected cells, rather than heterogeneous viral loads in each infected cell (22). Accordingly, the CHL carrier state likely identifies the persistent presence of an increased number of EBV-infected cells that infers some deficiency in EBV control. However, very few studies to date have attempted to address what in the specific host response permits the CHL carrier state or why risk of progression appears to vary in different organ recipients.

### What factors contribute to the development of the CHL carrier state?

Biologic explanations of why some SOT recipients become CHL carriers and others do not remain a mystery. The presence of EBV in the peripheral blood is an expected outcome of primary EBV infection. Previously healthy individuals with acute EBV-associated mononucleosis have been shown to have EBV-infected B cells in the peripheral blood for weeks to years after resolution of symptoms, though at lower load levels and shorter durations compared to pediatric CHL carriers (23). Children developing primary EBV infection following SOT tend to follow one of three EBV load patterns: (1) transient acute elevation of EBV load with subsequent clearance; (2) transient acute elevations with subsequent persistence of chronic low levels of EBV load; or (3) transient elevations of EBV load with eventual, but variable, progression to the CHL carrier state. Multiple biological components likely contribute to a child developing one of the specific EBV load patterns, but data are limited. Some inferences can be drawn from observational reports and a small number of translational studies. These inferences may include net state of immunosuppression, young age at the time of primary EBV infection, route of transmission, and inoculum. Each of these inferences will be discussed separately in the paragraphs below.

The presence of immune suppression at the time of primary EBV infection appears to be an important predictor of development of the CHL carrier state, highlighting the key role of the initial host immune response in establishing long-term control of the EBV load in the peripheral blood. However, being immunosuppressed alone does not predict progression to the CHL state, as only some pediatric SOT recipients become CHL carriers after primary infection and EBV seropositive pediatric intestinal transplant recipients become CHL carriers as frequently as those who are seronegative prior to transplant. Why this happens in children undergoing intestinal transplant and not those undergoing other types of SOT recipients remains unknown. Other patients who are substantially immunosuppressed, including stem cell transplant recipients also develop very high EBV loads, though less is known about whether they will maintain a chronic asymptomatic state.

Age is another potential contributor to development of the CHL carrier state in pediatric SOT recipients. Multiple studies have found that age less than five years is an independent risk for CHL development, although the mechanism remains uncertain (19). Studies of otherwise healthy infants with primary EBV infection provide some potential explanations for this phenomenon. For example, healthy infants living in an area endemic for Burkitt's lymphoma are known to develop persistent EBV loads following primary EBV infection for months or even a few years (24). Infants with primary EBV infection rarely develop infectious mononucleosis, likely due to immature T-cell response against EBV-infected cells. These examples suggest an insufficient immune response to EBV infection in a healthy infant, the impact of which may be amplified in an already immunosuppressed SOT infant/young child recipient. The immunosuppression for organ transplantation on top of the natural immature immunity may lead to the development of the CHL carrier state in young pediatric SOT recipients.

Finally, the route and inoculum of initial EBV transmission may contribute to the development of CHL carrier state. Most primary EBV infections in EBV-mismatched recipients are thought to be donor-derived through the graft, representing a non-physiological route of infection. A study using immunocompetent humanized mice has shown that high inoculum of EBV to establish primary infection results in high and persistent EBV loads in blood and a high incidence of lymphoproliferative disorders, as compared to low EBV inoculum (25). This may explain differences in progression to the CHL carrier state in those with infection early as opposed to late after transplant as the intensity of immunosuppression will likely be substantially lower later after transplant.

### What is the host immune response related to CHL?

Expansion of EBV-infected cells is primarily controlled by cytotoxic T cells and natural killer (NK) cells in immunocompetent hosts (26). It is also known that some EBV-encoded proteins are highly immunogenic and usually provoke strong T-cell responses. Calcineurin inhibitors are frequently used as baseline immunosuppression in SOT patients and will suppress the T-cell response. Therefore, insufficient T-cell response, including failure of T-cell proliferation, failure of functional effector T-cell development and loss of T-cell cytotoxicity are associated with development of the CHL carrier state. Several reports have shown the presence of exhaustion signatures in CD8+ T cells and NK cells among SOT recipients with EBV loads and these exhaustion signatures are accompanied by the presence of co-inhibitory molecules such as programmed death 1 (PD-1) (27). However, expression of these markers may vary amongst CHLs, perhaps dictated in part by organ recipient type, and may correlate with risk of progression to PTLD. The reported greater risk of progression to PTLD in pediatric heart CHLs may be explained by the observation of an increase of terminally exhausted T cells compared to liver and kidney transplant recipients with CHL (21). There may be other immunologic features such as the use of lymphoablative therapy and history of thymectomy contributing to the development of CHL carrier state, but little is known of their direct impact on EBV loads.

## Does the presence of viral set-points contribute to the development of the CHL carrier state?

Observations from HIV-infected patients and otherwise healthy individuals with EBV infection suggest the presence of viral set-points (some low and some high) for each EBV-infected patient. The set-point for a given patient is thought to reflect the physiologic host-virus equilibrium state in that individual, and once established it appears to be stable for years, at least in the HIV population (2). A wide range of set points occur, from very low load levels that are below the detection limit with available NAAT assays to very high as seen in some patients with HIV (2) and other immunosuppressed patients including SOT recipients and the presence of the CHL carrier state is associated with a high viral load set point compared to low or no load. The components of the immune response determining the set point for any given patient remain unknown. Among SOT recipients, persistent EBV loads were observed over long time periods despite efforts to reduce the immunosuppression in these patients and in some children even after stopping immunosuppression therapy (16). At present, while we do not know the precise mechanism that establishes and maintains a given set-point, an elevated set-point appears to be determined by the attenuated primary immune response against EBV-infected B cells under immunosuppression resulting in a larger number of circulating, latently infected cells. Notably, these latently infected cells only express a few EBV encoded proteins instead of highly antigenic ones, thus these EBV infected cells are known to be off-target from T-cell immune responses.

### Which compartment should you sample when monitoring serial EBV PCR tests?

While the recently published International Pediatric Transplant Association (IPTA) consensus guidelines support the use of either WB or plasma use for EBV DNA monitoring, the guidelines state that the use of WB for surveillance may be preferred to maximize sensitivity for early detection of EBV DNA (2). Plasma testing is thought to be less sensitive in detecting early EBV infection, but more specific in detecting disease and monitoring of treatment response (2). While we support the IPTA guidelines which recommend using whole blood EBV load to inform titration of immunosuppression as a prevention strategy against EBV/PTLD (28), the best compartment for monitoring a CHL carrier has yet to be determined. The predictive value of sequential measurement of plasma-based EBV loads in EBV CHL carriers is unknown because previous studies utilized PBL or whole blood-based EBV loads to define CHL. Future studies are needed to identify comparative predictive values for measurement of plasma-based vs. whole blood EBV loads in CHL.

### Who should be monitored?

The risk factors for developing either CHL or PTLD have been addressed in multiple observational and retrospective studies (4–8, 19). Recipients who are EBV naïve prior to transplant and who are younger than five years of age are at high risk for the development of both EBV/PTLD and CHL carrier state (2, 7, 19). Therefore, the current guidelines recommend that patients at high risk for PTLD should be regularly monitored for the first-year post-transplant. In general, monitoring is most frequent in the first 3–6 months post-transplant and then becomes less frequent over time. While monitoring is typically continued beyond the first year after transplant, the frequency is typically reduced to only 2–4 times per year in those with persistent negative or stable positive loads. Evidence identifying the optimal frequency of monitoring for children following primary infection, resolution of disease, or while meeting the definition of the CHL carrier is lacking. While a IPTA EBV/PTLD diagnostic guideline provides commentary, specific evidence-based recommendations supporting specific algorithms for these patients are not provided.

### What are the clinical responses to EBV DNAemia and CHL?

Reduction of immune suppression should be considered for patients with rapidly increasing EBV loads, those with newly recognized high loads, and those meeting the definition of the CHL carrier state. Although this approach does not necessarily result in the clearance of EBV loads, experience suggests that it often helps stabilize the load at a lower level. We typically recommend discontinuing or markedly reducing secondary agents, such as mycophenolate mofetil, and reducing calcineurin inhibitors by 25–50 percent, as long as clinical condition allows. Newly recognized CHL carriers should be carefully evaluated for signs and symptoms of EBV disease including PTLD. At present, data confirming the outcome of this approach in EBV CHL carriers is limited and difficult to assess due to the lack of uniformity in EBV assays, types and doses of immunosuppressive agents, and management in each transplant center. A few studies from a single center highlighted differences in organ-specific risk suggesting that different types of SOT may require different management (7, 21). Reports documenting retrospective experience and ideally prospective outcomes of management protocols are needed to optimize our approach to these patients as the precise balance between reducing immunosuppression to prevent PTLD and the risk of rejection is unclear.

### How should CHL carriers be followed?

The frequency of EBV load monitoring is typically increased once the CHL carrier state is established. This period of more intense monitoring helps to ascertain the dynamics of patient EBV loads and confirm that they are not rising rapidly. We typically monitor EBV loads every 3–4 months in recently identified CHL carriers. The interval between monitoring may be prolonged for those demonstrating stable high loads (within 0.5 logs) over time, suggesting establishment of a set-point equilibrium.

Pediatric SOT recipients meeting the definition of a CHL carrier state are often already on minimal immunosuppressive therapy. Thus, they may be at risk for both progression to PTLD and development of rejection. Accordingly, EBV CHL carriers should be carefully followed for signs and symptoms of both PTLD and rejection as they may overlap. Typical signs and symptoms of PTLD include fever, malaise, inadequate weight change, night sweats, adenopathy, diarrhea, and abnormal liver injury tests, while symptoms of rejection also include fever, malaise, and other non-specific symptoms. While the CHL carrier state resolves spontaneously in many children over time, a rebound of EB viral load can occur. The meaning of this phenomenon is not fully understood but again, this does not necessarily predict progression to EBV/PTLD.

### Future research

Additional investigations are needed to improve our understanding of the CHL carrier state and the host immune response to EBV in SOT recipients. The following questions are among many deserving of study:

1) What metrics should be used to inform progressive reduction in immune suppression for CHL carriers?
2) Is there a role for use of pre-emptive anti-CD20 antibody therapy in CHL carriers?
3) Is there a role for EBV-specific T-cell therapy in CHL carriers?
4) Are there biological markers that can better inform risk assessment and management of the CHL carrier state?

## Conclusions

The introduction of clinical use of EBV load monitoring in the peripheral blood represented an important translation of the biology of EBV infection to the clinical management of pediatric SOT recipients. While data support sequential measurement of EBV loads in the prevention and diagnosis of early EBV/PTLD, use of this approach has identified a cohort of pediatric SOT recipients manifesting an asymptomatic EBV CHL carrier state. While the outcome of the CHL carriers varies amongst organs, most will not progress to EBV/PTLD, and some resolve the carrier state albeit often after a prolonged period of time. The biological factors associated with the development of the CHL carrier state and optimal management of such patients remain to be defined. While this manuscript provides current recommendations, more basic, translational, and clinical data is needed to optimize our understanding and approach to this condition.
